# The Influence of Music Preference on Countermovement Jump and Maximal Isometric Performance in Active Females

**DOI:** 10.3390/jfmk8010034

**Published:** 2023-03-14

**Authors:** Rebecca R. Rogers, Tyler D. Williams, Emma B. Nester, Grace M. Owens, Christopher G. Ballmann

**Affiliations:** 1 Center for Engagement in Disability Health and Rehabilitation Sciences (CEDHARS), School of Health Professions, University of Alabama at Birmingham, 3810 Ridgeway, Birmingham, AL 35209, USA; 2 Department of Kinesiology, Samford University, Birmingham, AL 35229, USA

**Keywords:** motivation, force, preferred, non-preferred, rate of perceived exertion

## Abstract

Previous studies have shown that listening to preferred music during resistance and endurance exercises improves performance. However, it is unknown if these phenomena translate to short-duration explosive exercises. The purpose of this study was to investigate the influences of preferred and non-preferred music on countermovement jump (CMJ) performance, isometric mid-thigh pull (IMTP), and psychological responses to music during explosive movements. Physically active females (age 18–25) volunteered to take part in the study. In a counterbalanced, crossover design, participants completed three trials: (1) no music (NM), (2) non-preferred (NP), and (3) preferred (PV) music. Participants completed three maximal IMTP tests on a force-plate-equipped IMTP apparatus with an immovable bar. Attempts lasted 5 s and were separated by 3 min of rest. Furthermore, participants completed three single maximal CMJ attempts separated by 3 min of rest on force plates. All attempts were averaged for analysis. At the commencement of IMTP and CMJ testing, participants were asked to rate how motivated and psyched up they felt during the exercise portion using a visual analog scale. For isometric performance, listening to PM resulted in increased peak force (*p* = 0.039; d = 0.41) and rate of force development at 200 ms (*p* = 0.023; d = 0.91) compared with NP. For CMJ, there were no differences between conditions for jump height (*p* = 0.912; η^2^ = 0.007) or peak power during the propulsive phase (*p* = 0.460; η^2^ = 0.003). Levels of motivation were significantly higher with PM compared with NM (*p* < 0.001; d = 2.3) and NP (*p* = 0.001; d = 2.0). Feelings of being psyched up were significantly higher with PM compared with NM (*p* < 0.001; d = 4.2) and NP (*p* = 0.001; d = 2.8). Findings suggest that preferred music enhances isometric strength and increases motivation and feelings of being psyched up. Thus, PM may be used as an ergogenic aid during short-duration maximal-effort activities.

## 1. Introduction

The use of music as a motivating factor and ergogenic aid during exercise has been well supported in multiple exercise modalities including sprint, endurance, and resistance exercise [1]. Underlying mediators of performance improvements may be physiological (i.e., heart rate, hormones), psychological (i.e., motivation, enjoyment), or psychophysiological (i.e., rate of perceived exertion) which may act independently or synergistically [2]. However, responses to music are highly individualized, necessitating careful consideration of factors that may alter the efficacy of music implementation for performance. Recent studies have established that individual music preference largely mediates performance enhancement [2,3,4,5,6]. However, the characterization of how music preference influences strength- and power-based exercise remains incomprehensive.

Preferred music genre has been shown to improve power-based metrics in both sprint and resistance exercise [2,3,5,6,7,8]. In particular, Ballmann et al. showed that listening to preferred music during exercise improved power output and barbell velocity during bench press at 75% of maximal strength versus non-preferred [5]. Bolstering this, a follow-up study in resistance-trained males showed increased power output during upper-body resistance exercise when preferred music is played immediately prior to giving effort [7]. For sprinting, Meglic et al. showed increased mean power and total work during repeated sprints with preferred music in Division I female collegiate athletes [6]. Despite this, conflicting reports on preferred music and power development during exercise exist which may in part be due to differences in intrinsic characteristics of the music or intensity/duration of exercise. Thus, a more diverse understanding of how preferred music may influence various power-based exercises is needed to form comprehensive conclusions. 

Mechanistic determinants of responses to exercise while listening to preferred music have recently been suggested to primarily manifest in psychological and psychophysiological domains [3,4,5,6,8]. Listening to preferred music has been repeatedly shown to result in increases in motivation [3,4,5,6]. Interestingly, there have been reports that increases in motivation while listening to preferred music may be more pronounced in females than males, suggesting that females may benefit from changes in psychological factors with preferred music more than male counterparts [8]. Motivation may lead to increased drive and effort, supporting the notion that underlying ergogenic benefits may be dependent on increases in feelings of motivation [9]. Dissociation has also been shown to increase with preferred music which is reflected in decreases in ratings of perceived exertion [4]. This is likely achieved by shifting attention away from discomfort during exercise and toward the external stimuli of music. Indeed, multiple investigations have shown lower ratings of perceived exertion during repeated sprints with preferred music, suggesting potent psychophysiological modulation [4,6].

To date, knowledge of how music preference influences short explosive exercise is limited. Recently, Gavadana et al. reported that self-selected stimulatory music did not enhance jumping performance in male volleyball players [10]. Other studies in resistance-trained males similarly found no differences in jump height while listening to self-selected music [11]. However, these studies did not account for music preference, which is an important distinction as previous evidence has shown that non-preferred music may worsen responses in the absence of benefit from preferred music [2]. Furthermore, females have been suggested to respond to self-selected music more favorably than males during repeated sprints [8]. Since females have been excluded from previous investigations, this may partially explain the lack of performance improvements. Thus, the purpose of this study was to investigate the influences of preferred and non-preferred music on countermovement jump (CMJ) performance, isometric mid-thigh pull (IMTP) metrics, and psychological responses to music during explosive movements in active females. Since preferred music has been shown to improve power development during repeated resistance and sprint exercises compared with non-preferred [2,5,6,7,12], we hypothesized that preferred music would result in improved CMJ and IMTP performance while non-preferred music would not result in any ergogenic benefit. Furthermore, given previous reports of robust changes in feelings of motivation which have been suggested as pivotal for performance changes with preferred music [3,4,5,6,7], we hypothesized that preferred music would increase feelings of motivation and being psyched up while non-preferred would not. 

## 2. Materials and Methods

### 2.1. Study Design

In a counterbalanced, crossover manner, female participants completed three different exercise sessions, each with a different experimental condition: no music (NM), non-preferred music (NPM), and preferred music (PM). For each condition, participants completed CMJ and IMTP tests in a counterbalanced manner where each test consisted of three maximal attempts. After each test, motivation and feelings of being psyched up were documented. Each visit was separated by a minimum of 48 h. Direct comparisons between NM, NPM, and PM were made for peak force, rate of force development, jump height, peak velocity, motivation, and feelings of being psyched up. Prior to any data collection, verbal and written informed consent was obtained from each participant. All experimental procedures were conducted in accordance with the Declaration of Helsinki (2013) and approved by the Samford University Institutional Review Board (EXPD-HP-22-SUM-2; June 2022).

### 2.2. Participants

Adequate sample size was determined using an a priori power analysis with G*power statistical software (G*power V 3.1.9.4). A previous investigation from our group in 2020 showed increases in relative power output during exercise while listening to PM compared with NPM and NM with an effect size of f = 0.71 [12]. To calculate the minimum sample size needed, the following parameters were used: test = ANOVA (repeated measures), f = 0.71, α = 0.05, 1-β = 0.8, corr = 0.5. This equated to a minimum sample size of *n* = 9. In order to have comparable sample sizes to previous investigations [2,3,4,5,6,7,12], 12 physically active females (20.9 yrs ± 0.3, 59.8 kg ± 8.0, 163.8 cm ± 5.0) were recruited to participate. To be considered physically active, participants had to report attainment of at least 150 min/wk of moderate-intensity exercise [13]. Other inclusion criteria included being free from injuries that limited exercise activity six months prior to participation and no self-reported diagnosis of hearing loss. To screen for the safety of exercise, participants also completed a physical activity readiness questionnaire (PAR-Q). Participants were asked to refrain from vigorous activity 24 h prior and from alcohol, caffeine, and nicotine 12 h prior to each testing session [12,14]. Participants were unaware of any experimental hypotheses.

### 2.3. Music Preference and Familiarization

Upon the arrival of the first session, participants completed a music preference questionnaire as previously described by our lab [3,4,5,6,12]. Briefly, participants were given a ranking list of different music genres including rap/hip hop, country, pop, dance electronic, and rock and roll. Genres were rated from most preferred (highest rank) to least preferred (lowest rank). For the PM music condition, participants chose any song from their most preferred genre as long as it had a tempo of ≥120 bpm. In an attempt to eliminate any participant preference during the NPM condition, researchers selected a tempo-matched song from the participant’s least favorite genre. Songs were looped/repeated for testing. Music was listened to through Bluetooth headphones (Apple Inc., Cupertino, CA, USA) at the same volume level for all participants to where participants could hear shouted commands from researchers.

Before beginning each test, participants were familiarized and given practice attempts to ensure proper form and understanding of the exercise. To achieve this, researchers first demonstrated each test’s proper technique and progression. Then, percent effort was ramped up in such a way to where participants completed one practice attempt at 50%, 75%, and also 100% effort interspersed by 2 min of rest. The form was corrected throughout these attempts as needed and, if necessary, demonstration of proper technique was repeated.

### 2.4. Testing Procedures

Upon arrival, height and weight were documented. A brief warm-up was completed on a cycle ergometer at 50 watts for 5 min. In a counterbalanced manner, participants completed three attempts of IMTP and CMJ testing. For music conditions, the commencement of music was initiated immediately prior to the first attempt and remained playing until the end of the last attempt. The IMTP was used to determine maximal isometric force and rate of force development. The IMTP protocol followed the testing procedures developed by Comfort et al. [15]. Briefly, participants stood on an IMTP rig (Samson; Las Cruces, NM, USA) equipped with a force platform system (Hawkin Dynamics, USA) which had been previously validated [16]. The IMTP apparatus was situated with an immovable bar that was adjusted to the appropriate height of the thigh to position the participant in the second pull position of the clean. Participants were positioned with an erect torso, knees, and hips slightly flexed, elbows extended, and hands placed in a pronated position outside the thigh. To prevent grip slip, wrist wraps were used to anchor participants’ hands to the bar. The participant’s feet and hand placement were recorded and used to reproduce IMTP position between sessions. Participants were instructed to assume the IMTP position without pulling up on the bar. When prompted, participants maximally pulled upward and with driving force originating from their legs for a total effort of 5 s. This was repeated a total of three times with 3 min of rest in between attempts. Peak force (N) and rate of force development at 200 ms (N·s^−1^) were recorded for each attempt. Following a 5 min rest, participants completed maximal CMJ testing. Briefly, participants stood on a force platform system (Hawkin Dynamics, Westbrook, Maine, USA) with hands on their hips and feet shoulder-width apart. When prompted by researchers, participants completed a single maximal CMJ where they squatted to a knee joint angle of approximately 90 degrees and jumped “as high as possible”. Participants had to maintain their hands on their hips at all times and reach a proper depth for it to be considered a successful attempt. This was repeated a total of three times with 3 min of rest in between attempts. Jump height (m) and peak velocity (m·s^−1^) were recorded from the CMJ. All three attempts for each test were averaged for analysis. Following each test, feelings of motivation and being “psyched up” were collected via a visual analog scale (VAS), as previously described by our lab [3,4,5,6,7,8,12,17,18]. For each VAS, participants marked their subjective feelings of motivation or being “psyched-up” on a 100 mm line, whereby 0 indicated the absence of the feeling and 100 indicated the strongest feeling. All performance and psychological values were recorded after each test and averaged together for each condition for analysis.

### 2.5. Statistical Analysis

All analytics were completed using Jamovi software (Version 0.9; Sydney, Australia). Confirmation of normality was determined via the Shapiro–Wilk method. To confirm the reliability of measurements between attempts, intraclass correlations (ICC) were determined for IMTP (ICC = 0.89; lower 95% CI = 0.74; upper 95% CI = 0.96) and CMJ (ICC = 0.94; lower 95% CI = 0.85; upper 95% CI = 0.96) using peak force and jump height, respectively. For all comparisons, a 1 × 3 repeated measures ANOVA [test × condition] was used to determine main effects. A Bonferroni–Holm post-hoc test was used to determine differences between means. Estimates of effect size for main effects were calculated using eta squared (η^2^) and interpreted as: 0.01—small; 0.06—medium; ≥0.14—large [19,20]. All data are presented as mean ± standard deviation (SD). Significance was set at *p* ≤ 0.05 a priori.

## 3. Results

**Figure 1:**
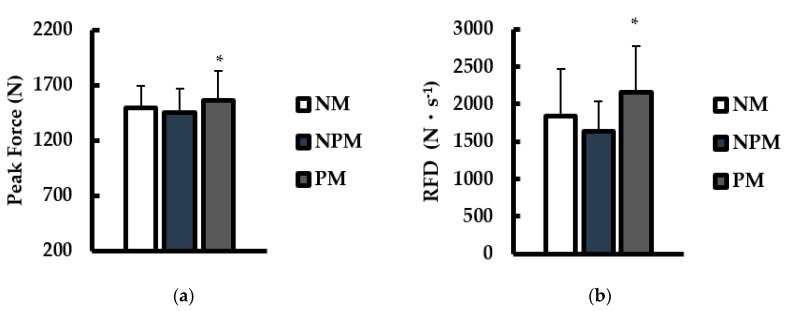
Comparisons of (**a**) peak force (N) and (**b**) rate of force development (at 200 ms; N·s^−1^) during maximal isometric mid-thigh pull tests between no music (NM; white), non-preferred music (NPM; blue), and preferred music (PM; grey) conditions Data are presented as mean ± SD. * indicates significantly different than NPM (*p* < 0.05).

Peak force (N) and rate of force development at 200 ms (RFD; N·s^−1^) during IMTP testing are shown in Figure 1. For force (Figure 1a), there was a main effect for condition (*p* = 0.039; η^2^ = 0.109). Peak force was significantly higher with PM versus NPM (*p* = 0.033; d = 0.41). For RFD (Figure 1b), there was a main effect for condition (*p* = 0.035; η^2^ = 0.201). RFD was significantly higher with PM versus NPM (*p* = 0.033; d = 0.91).

**Figure 2:**
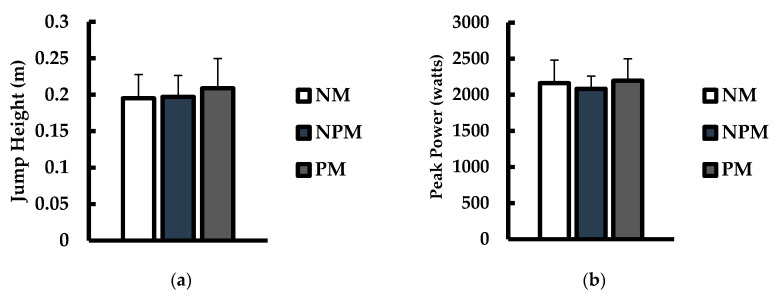
Comparisons of (**a**) jump height (m) and (**b**) peak power (watts) during the propulsive phase during countermovement jump tests between no music (NM; white), non-preferred music (NPM; blue), and preferred music (PM; grey) conditions. Data are presented as mean ± SD.

Jump height (m) and peak power during the propulsive phase (watts) for CMJ testing are shown in Figure 2. For jump height (Figure 2a), there were no differences between conditions (*p* = 0.375; η^2^ = 0.007). Furthermore, no differences in peak power (Figure 2b) were noted between conditions (*p* = 0.460; η^2^ = 0.003).

**Figure 3:**
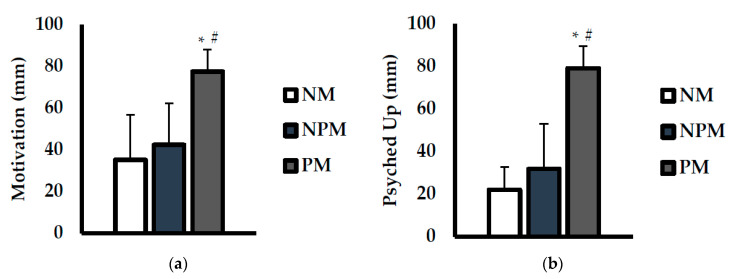
Comparisons of (**a**) motivation and (**b**) feelings of being psyched up during testing between no music (NM; white), non-preferred music (NPM; blue), and preferred music (PM; grey) conditions. Data are presented as mean ± SD. * indicates significantly different than NPM (*p* < 0.05). # indicates significantly different than NM (*p* < 0.05).

Feelings of motivation (mm) and being psyched up (mm) are shown in Figure 3. For motivation (Figure 3a), there was a main effect for condition (*p* < 0.001; η^2^ = 0.752). Motivation was significantly higher with PM compared with NM (*p* < 0.001; d =2.3) and NPM (*p* < 0.001; d = 2.0). For feelings of being psyched up (Figure 3b), there was also a main effect for condition (*p* < 0.001; η^2^ = 0.491). Feelings of being psyched up were significantly higher with PM compared with NM (*p* < 0.001; d = 4.2) and NPM (*p* < 0.001; d = 2.8).

## 4. Discussion

Individual preference has been well described to mediate the ergogenic potential of music [2]. Specific to the current investigation, preferred music has been suggested to improve power output during resistance and sprint exercises [2,5,6]. However, no studies to date have investigated whether music preference influences short, maximal efforts during exercise, leaving a gap in knowledge as to what particular actions may benefit from the implementation of preferred music. The purpose of this investigation was to determine if preferred and non-preferred music influence performance and psychological responses during explosive movements (i.e., CMJ, IMTP) in active females. Current findings suggest that listening to PM results in improved isometric force metrics compared with NPM. However, no performance improvements were seen for jump height or peak velocity during CMJ tests. Feelings of motivation and being psyched up were markedly higher with PM compared with NPM and NM. While more study is needed, these data present supporting evidence that PM may be ergogenic during maximal isometric exercise in physically active females to which alterations in motivation and being psyched up may be primary contributors to performance enhancement.

Performance during IMTP testing is reflective of the rate and magnitude of force generation capabilities. Currently, both peak force and rate of force development were enhanced with PM compared with NPM. This partially supports previous findings of increased power during bench press and sprinting with PM versus NPM, albeit current IMTP testing lacked a velocity component [2,5,6]. Indeed, our lab has reported increased power output and barbell velocity during explosive bench-press exercise in resistance-trained males [5]. The underlying reasons for performance enhancement with PM are not clear from the present data alone but may be related to PM inducing a greater stimulative effect both from physiological and psychological domains. Music has been shown to increase anticipatory responses and sympathetic stimulation during exercise [21]. For example, Yamamoto et al. showed exacerbated catecholamine responses to exercise in males with music which could accentuate muscle contractile function [21]. While speculative, PM may have elicited a greater sympathetic response allowing for greater effort and increased muscular force production compared with NPM. Psychologically, current increases in feelings of being psyched up may have mediated performance improvements with PM. Arousal and vigor have been suggested to be heightened with PM in healthy individuals [1,2]. Heightened feelings of arousal and being psyched up may have led to increased task preparedness and the ability to give maximal effort. Indeed, self-selected music has been shown to induce improvements in isometric knee extensor strength with increased feelings of arousal in males [22]. Thus, improvements in force-generating capabilities with PM may be due to independent or synergistic actions of physiological or psychological factors, but more research is needed to detail contributing mechanisms. 

Counter to our hypothesis, metrics from CMJ remained largely unchanged irrespective of music condition. This supports previous negative findings employing self-selected music [10,11]. Effort during CMJ is extremely short in duration compared with exercise employed in previous investigations. It is possible that the time period of effort during CMJ is too short to be enhanced by music. The effectiveness of music as a performance enhancer has been reported to peak at later points during exercise and/or during times of fatigue. Since the CMJ test was short and non-fatiguing, benefits from PM may have not been realized under the current protocol. Furthermore, CMJ performance is highly influenced by the stretch-shortening cycle of the active musculature [23]. At this time, we are unaware of a rationale for PM to instill benefits to the stretch-shortening cycle which may indicate music has less potential to optimize jumping or countermovements highly reliant on stretch-shortening. Taken together, listening to PM does not appear to be an effective strategy to enhance single jump performance, although a further study on repeated or fatigue-inducing jumping is warranted. 

The potent ability of PM to increase motivation has been well-established in both males and females [2,3,4,5,6,12]. Current increases in motivation support these findings and suggest that the motivational effects of PM are maintained throughout single maximal effort power-based movements. Furthermore, feeling psyched up, which is reflective of arousal levels, was significantly higher while listening to PM. Subjective benefits from PM have also been confirmed physiologically via functional magnetic resonance imaging whereby listening to favorite music initiates signaling cascades in the nucleus accumbens and ventral striatum in the brain [24]. In particular, these regions of the brain are largely responsible for the control of reward pathways and motivational drive. Since increases in motivational drive often lead to increased effort and arousal, it is probable that PM induced similar psychophysiological changes which may have underpinned ergogenic benefits during the IMTP test. Despite no differences in feelings of motivation or being psyched up between IMTP and CMJ tests (data not shown), no performance enhancement was seen with PM during CMJ testing. This could in part be due to the intensities of the tests themselves. While both tests are maximal in nature, IMTP requires longer effort with greater engagement of muscle mass compared with CMJ. Previous evidence has established that PM results in greater dissociation during strenuous activities [2,4]. In addition, external shifting of focus, such as that to music, may also improve high-intensity ballistic exercise performance in active populations [25]. Thus, PM music may have had a larger opportunity to have an influence on IMTP compared with CMJ tests because of the longer and more intense efforts that the test requires. More studies on specific movements and exercises that are susceptible to performance enhancement with music are needed. 

The current study provides novel findings of how music preference influences strength and power performance. Nevertheless, limitations still existed. First, these outcomes and tests are more centered toward strength athletes, given the importance of power developed in their sport. The current sample was physically active but not necessarily strength trained. Future studies should implement a more strategic sampling of well-trained strength athletes or other types of competitors. A more in-depth look into additional performance variables other than those measured currently may provide greater insight into mechanisms for performance changes and how training status may affect them. Furthermore, only females were included which may not be representative of the general population, especially since females may react to music differently during exercise than their male counterparts [8]. While the current study did not control for menstrual cycle phase, previous evidence has shown that listening to music during high-intensity anaerobic exercise is equally as effective across all menstrual cycle phases [26]. Notably, females are drastically understudied in these contexts which makes a more intentional study of this population necessary to improve knowledge in this area [27,28]. Despite these limitations, current data provide advancement to the knowledge of music preference and exercise performance all while emphasizing the need for more investigations into how different aspects of music may be preferred and how that might alter performance differently.

## 5. Conclusions

In conclusion, the present findings showed that listening to PM resulted in superior performance in physically active females during maximal isometric testing, but not vertical jump testing, as compared with NPM. Psychological measures of motivation and feeling psyched up were heightened and may in part contribute to changes in performance. From a practical standpoint, music played in communal settings (i.e., gyms, locker rooms, sporting events, etc.) is often played on overhead speakers outside of the control of the individual. Current data suggest that athletes or competitors should ensure that the music they are listening to is of their own preference during single maximal effort exercise. In particular, this may be especially useful during competition in athletes engaging in short explosive movements, such as sprinters, jumpers, and powerlifters. Furthermore, individuals seeking to improve their motivation or who are looking to psych themselves up in anticipation of giving maximal effort may benefit from utilizing PM. Psychological improvements in motivation may lead to greater effort and physical drive, but more study is needed to fully characterize psychological responses to PM.

## Data Availability

All data are freely available within this manuscript.

## References

[1] Terry P.C., Karageorghis C.I., Curran M.L., Martin O.V., Parsons-Smith R.L. (2020). Effects of music in exercise and sport: A meta-analytic review. Psychol. Bull..

[2] Ballmann C.G. (2021). The Influence of Music Preference on Exercise Responses and Performance: A Review. J. Funct. Morphol. Kinesiol..

[3] Ballmann C.G., Cook G.D., Hester Z.T., Kopec T.J., Williams T.D., Rogers R.R. (2020). Effects of Preferred and Non-Preferred Warm-Up Music on Resistance Exercise Performance. J. Funct. Morphol. Kinesiol..

[4] Ballmann C.G., Maynard D.J., Lafoon Z.N., Marshall M.R., Williams T.D., Rogers R.R. (2019). Effects of Listening to Preferred versus Non-Preferred Music on Repeated Wingate Anaerobic Test Performance. Sports.

[5] Ballmann C.G., McCullum M.J., Rogers R.R., Marshall M.R., Williams T.D. (2021). Effects of Preferred vs. Nonpreferred Music on Resistance Exercise Performance. J. Strength Cond. Res..

[6] Meglic C.E., Orman C.M., Rogers R.R., Williams T.D., Ballmann C.G. (2021). Influence of Warm-Up Music Preference on Anaerobic Exercise Performance in Division I NCAA Female Athletes. J. Funct. Morphol. Kinesiol..

[7] Ballmann C.G., Favre M.L., Phillips M.T., Rogers R.R., Pederson J.A., Williams T.D. (2021). Effect of Pre-Exercise Music on Bench Press Power, Velocity, and Repetition Volume. Percept. Mot. Ski..

[8] Rhoads K.J., Sosa S.R., Rogers R.R., Kopec T.J., Ballmann C.G. (2021). Sex differences in response to listening to self-selected music during repeated high-intensity sprint exercise. Sexes.

[9] Barwood M.J., Weston N.J., Thelwell R., Page J. (2009). A motivational music and video intervention improves high-intensity exercise performance. J. Sport. Sci. Med..

[10] Gavanda S., Hosang T., Wagener S., Sönmez N., Kayser I., Knicker A. (2022). The Influence of Relaxing and Self-Selected Stimulating Music on Vertical Jump Performance in Male Volleyball Players. Int. J. Exerc. Sci..

[11] Biagini M.S., Brown L.E., Coburn J.W., Judelson D.A., Statler T.A., Bottaro M., Tran T.T., Longo N.A. (2012). Effects of self-selected music on strength, explosiveness, and mood. J. Strength Cond. Res..

[12] Karow M.C., Rogers R.R., Pederson J.A., Williams T.D., Marshall M.R., Ballmann C.G. (2020). Effects of Preferred and Nonpreferred Warm-Up Music on Exercise Performance. Percept. Mot. Ski..

[13] qRiebe D., Ehrman J.K., Liguori G., Magal M., American College of Sports Medicine (2018). ACSM’s Guidelines for Exercise Testing and Prescription.

[14] Dumar A.M., Huntington A.F., Rogers R.R., Kopec T.J., Williams T.D., Ballmann C.G. (2021). Acute Beetroot Juice Supplementation Attenuates Morning-Associated Decrements in Supramaximal Exercise Performance in Trained Sprinters. Int. J. Env. Res. Public Health.

[15] Comfort P., Dos’ Santos T., Beckham G.K., Stone M.H., Guppy S.N., Haff G.G. (2019). Standardization and methodological considerations for the isometric midthigh pull. Strength Cond. J..

[16] Brady C.J., Harrison A.J., Flanagan E.P., Haff G.G., Comyns T.M. (2018). A comparison of the isometric midthigh pull and isometric squat: Intraday reliability, usefulness, and the magnitude of difference between tests. Int. J. Sport. Physiol. Perform..

[17] Nixon K.M., Parker M.G., Elwell C.C., Pemberton A.L., Rogers R.R., Ballmann C.G. (2022). Effects of music volume preference on endurance exercise performance. J. Funct. Morphol. Kinesiol..

[18] Rogers R.R., Beardsley K.G., Cumbie P.E., Ballmann C.G. (2022). Ammonia Inhalants Enhance Psychophysiological Responses and Performance During Repeated High Intensity Exercise. Res. Q. Exerc. Sport.

[19] Fritz C.O., Morris P.E., Richler J.J. (2012). Effect size estimates: Current use, calculations, and interpretation. J. Exp. Psychol. Gen..

[20] Cohen J. (1988). Statistical Power Analysis for the Behavioral Sciences.

[21] Yamamoto T., Ohkuwa T., Itoh H., Kitoh M., Terasawa J., Tsuda T., Kitagawa S., Sato Y. (2003). Effects of pre-exercise listening to slow and fast rhythm music on supramaximal cycle performance and selected metabolic variables. Arch. Biochem. Biophys..

[22] Greco F., Rotundo L., Grazioli E., Parisi A., Carraro A., Muscoli C., Paoli A., Marcolin G., Emerenziani G.P. (2022). Effects of self-selected versus motivational music on lower limb muscle strength and affective state in middle-aged adults. PeerJ.

[23] Baechle T.R., Earle R.W. (2008). Essentials of Strength Training and Conditioning.

[24] Montag C., Reuter M., Axmacher N. (2011). How one’s favorite song activates the reward circuitry of the brain: Personality matters!. Behav. Brain Res..

[25] Wulf G., Shea C., Park J.-H. (2001). Attention and motor performance: Preferences for and advantages of an external focus. Res. Q. Exerc. Sport.

[26] Ghazel N., Souissi A., Chtourou H., Aloui G., Souissi N. (2022). The effect of music on short-term exercise performance during the different menstrual cycle phases in female handball players. Res. Sport. Med..

[27] Costello J.T., Bieuzen F., Bleakley C.M. (2014). Where are all the female participants in Sports and Exercise Medicine research?. Eur. J. Sport Sci..

[28] Cowley E.S., Olenick A.A., McNulty K.L., Ross E.Z. (2021). “Invisible sportswomen”: The sex data gap in sport and exercise science research. Women Sport Phys. Act. J..

